# Supplementary material for: ProteinPrompt: a webserver for predicting protein–protein interactions
Source: Bioinform Adv. 2022 Aug 17;2(1):vbac059. doi: 10.1093/bioadv/vbac059 (PMC9710678; doi:10.1093/bioadv/vbac059)
Supplement: vbac059_Supplementary_Data [file vbac059_supplementary_data.pdf]

ProteinPrompt: a webserver for the prediction of protein-protein-interactions - Supplementary material

Sebastian Canzler, Markus Fischer, David Ulbricht, Nikola Ristic,
Peter W. Hildebrand, René Staritzbichler

August 12, 2022

Note: the numbering of the sections in this document is identical to the numbering of the sections in the main manuscript to which the sections here refer.

2.1 Collecting data points

In total, we collected 41,482 positive protein-protein pairs with at most 50% sequence identity, while collecting 39,457 negative protein-protein pairs. The positive set contains 10,538 individual proteins and the negative set contains 13,304 individual proteins. A summary of the respective test and training sets can be seen in Table S1. An overview of the overlapping proteins among those four datasets is depicted in Figure S1. The following sections investigate the datasets in further detail.

Dataset	# protein pairs	# proteins
pos. training	36423	10175
neg. training	34640	13264
pos. testing	5059	4591
neg. testing	4817	6816

Table S1: Summary of individual protein pairs and number of unique proteins per dataset.

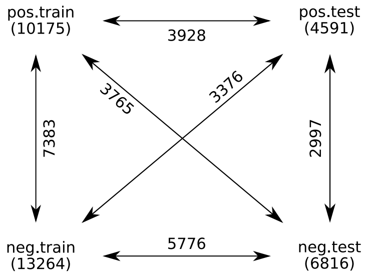

Figure S1: Number of overlapping proteins between different datasets. Numbers in parenthesis denote the number of unique proteins in each dataset.

Feature vector correlation	Sequence similarity 40%	Sequence Similarity 50%
> 0.8	0.00074%	0.0065%
> 0.9	0.0001%	0.00081%

Table S2: Percentage of protein pairs with a feature vector correlation above a threshold of 0.8 and 0.9.

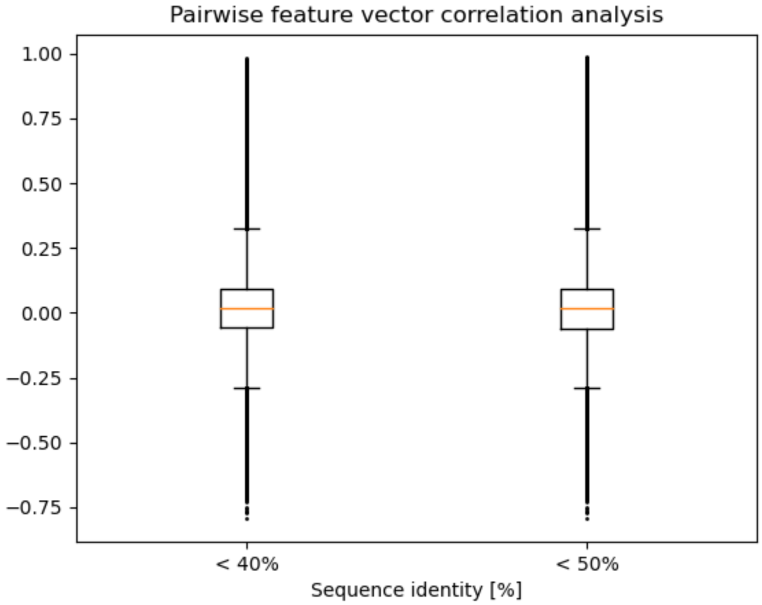

Figure S2: Boxplot representing the distribution of all pairwise feature vector correlations from protein data sets that have been filtered based on 50% and 40% sequence identity.

2.1.1 Analysis of the CD-hit threshold

To quantify the impact of a more stringent filtering of proteins with respect to their feature vectors, we calculated feature vetor correlations of pairwise proteins from a set of proteins with at most 50% sequence identity (as we used in our study) and a set of proteins with at most 40% sequence identity (max threshold offered by `CD-hit`).

Starting from a protein sequence database containing 31867 proteins (as described in section 2.1 of the manuscript), we ran `CD-hit` with cutoffs 50% and 40% and generated datasets containing 15728 and 13719 distinct protein clusters, respectively. For each of those clusters, we retrieved exactly one reference protein that shares at most 50% or 40% sequence identity to all other reference proteins, depending on the dataset.

Based on those two sets of reference proteins, we calculated all pairwise feature vector correlations within each dataset and plotted their distributions. For the set of 15728 proteins sharing at most 50% sequence identity, we calculated 123.677.128 pairwise feature vector correlations while calculating 94.112.340 pairwise correlations for the smaller dataset.

Although the more stringent filtering reduces the set of available proteins significantly (-13%) the correlation analysis of their respective feature vectors shows only a marginal improvement (see Table S2 and Figure S2).

	0 - 30%	30-40%	40-50%	50-60%	60-70%	70-80%	80-90%	90-100%
0 - 30%	177334846	126587	1405003	1315862	347567	73347	35897	152264
30-40%	124375	394	1192	1117	312	51	29	192
40-50%	1363827	1160	18244	15095	3560	715	319	1999
50-60%	1245961	1059	14776	17522	4001	672	279	2034
60-70%	337746	335	3555	4348	1566	251	88	716
70-80%	75261	54	757	727	242	118	22	195
80-90%	33803	36	332	330	72	30	19	61
90-100%	161494	234	2453	2410	827	212	45	65

Table S3: Training versus test data for binders.

2.2 Analysis of the data sets

In this section, we examine the relationship of sequence pairs in our datasets rather than of individual sequences as in the previous section. Because of the pairwise nature of binding, it is critical that different datasets do not contain similar pairs. A BLAST search was performed with an e-value cutoff of 100, creating pairwise alignments for all sequences in the datasets. Alignments with e-values larger than the threshold are considered insignificant or unrelated. Sequence pairs for which alignments were returned had similarities above 30%. Thus, in our depiction unaligned sequences with an e-value above 100 are subsumed into the region with similarity up to 30%. As we cannot distinguish anything below 30% similarity, we fused this into a larger bin. Above 30% there is a binning of 10%. However, in the range of 30 to 40% only few sequence pairs were aligned. Thus, we would consider this bin as rather unreliable.

To further investigate the influence of the remaining similarities, we removed in several steps all similarities above 60%, 50%, 40% and 30% between the training and test datasets. Table S6 shows the quality measures for the RF, Table S7 for the GNN. The data suggests that neither the RF nor the GNN suffer greatly from the removal of the remaining sequence similarity. Only the sensitivity and specificity of the RF appear to be affected by similarity cutoff of the datasets and more precisely by the size of the positive and negative training data sets (sizes are listed in Table Table S8). Interestingly, for RF the specificity increases with decreasing cutoff. This can be understood by the relative shift in size between binders (pos) and non-binders (neg). Naturally, the positive data has a higher propensity to be filtered due to similarity. For a 30% cutoff there are nearly twice as many negative data-points as positive (see Table S8). Thus, negative data will be overemphasized and this leads to an increase in specificity. This is because the random forest will try to minimize overall error rate, which in this case will result in an increase.

Since there is not much difference in the AUROC and AUPRC scores for the different datasets, we would argue that the two methods presented in the paper are robust to sequence similarity and also to its removal. Both algorithms seem to learn principles of the protein-protein-interactions beyond simple similarity detection. The magnitude of the changes in the overall performance currently observed could to some degree also be explained by the reduction in training data.

Figure S3 illustrates the calculation of similarities of data points. Figure S4 shows the similarities between the training and test data, Figure S5 shows similarities between entries within the training dataset. Figure S6 shows similarities between entries of the test dataset. Tables S3, S4, and S5 show the data that has been visualized in the those three figures, respectively.

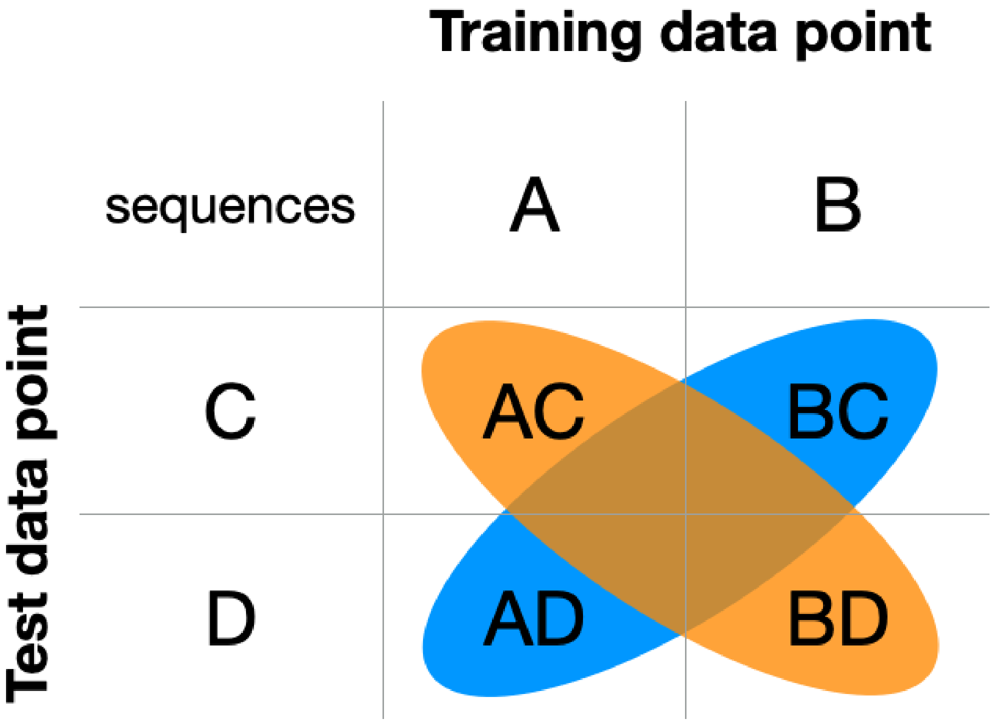

Figure S3: Example on how the similarities of datapoints are calculated. A datapoint (known binder or non-binder) from the training data may consist of sequences A and B. A datapoint from the test data may consist of sequences C and D. Underlaid in orange is the first possible combination: (AC,BD); in blue the second combination: (AD,BC). Similarities are calculated for all pairs individually: $S_1 = sim(A, C), S_2 = sim(B, D), S_3 = sim(A, D), S_4 = sim(B, C)$. Accordingly, the final similarity is $S = max(S1 + S2, S3 + S4)$.

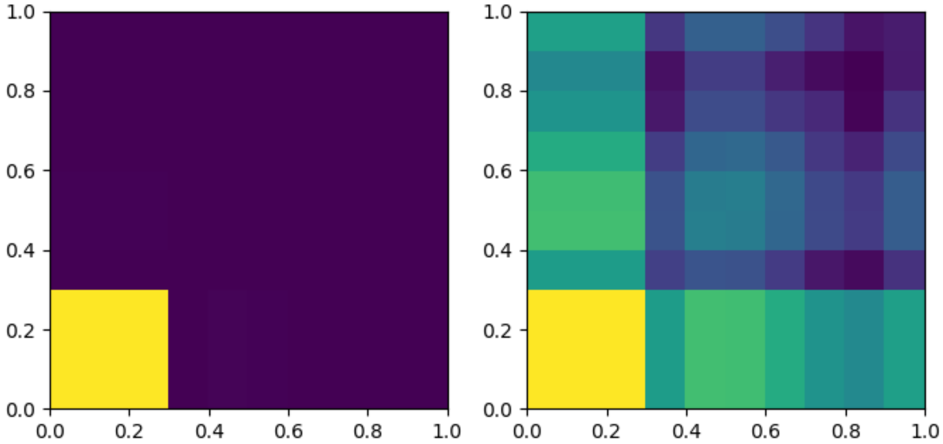

Figure S4: Redundancy analysis between binders in training and test data. Depicted is a 2D histogram of similarity between sequence pairs from binders of the training versus binding sequence pairs from the test dataset. The image on the right is in log-scale to visualize the background. See Table S3 for values. The image on the left is identical to Figure 1 in the main manuscript, except for the color scheme.

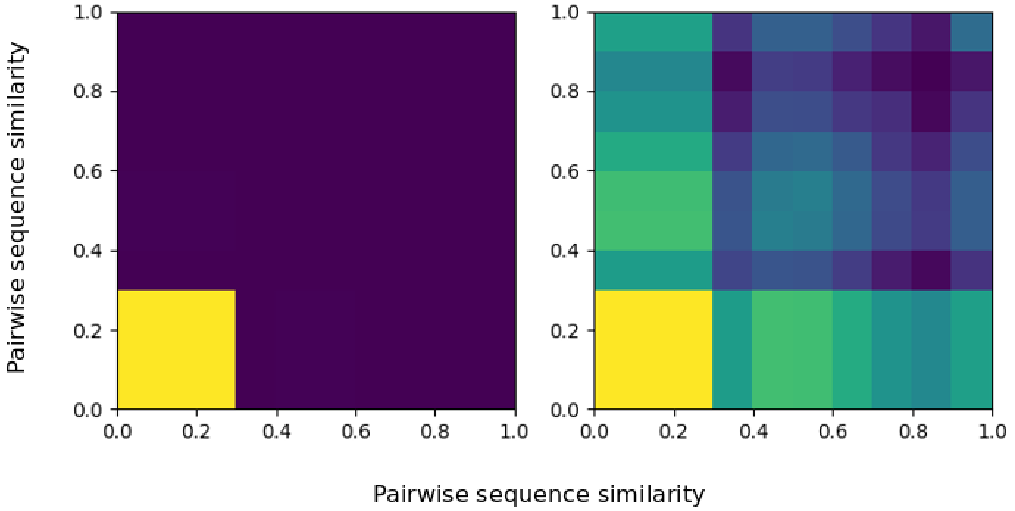

Figure S5: Redundancy analysis between binders in training data. See Table S4 for values.

	0 - 30%	30-40%	40-50%	50-60%	60-70%	70-80%	80-90%	90-100%
0 - 30%	1277793513	924217	9843509	9199670	2460137	521258	241930	1074056
30-40%	902712	3517	9117	8221	2330	464	193	1437
40-50%	9548046	9517	126468	102733	26506	5021	2123	15149
50-60%	8703841	8038	101608	126427	30691	5293	1955	15293
60-70%	2375120	2188	27561	32355	12064	1846	611	5691
70-80%	523609	476	5799	5629	1821	1107	178	1495
80-90%	227925	201	2394	2153	565	224	133	357
90-100%	1143738	1539	17066	17158	5817	1622	353	36939

Table S4: Training data cross similarities for binders.

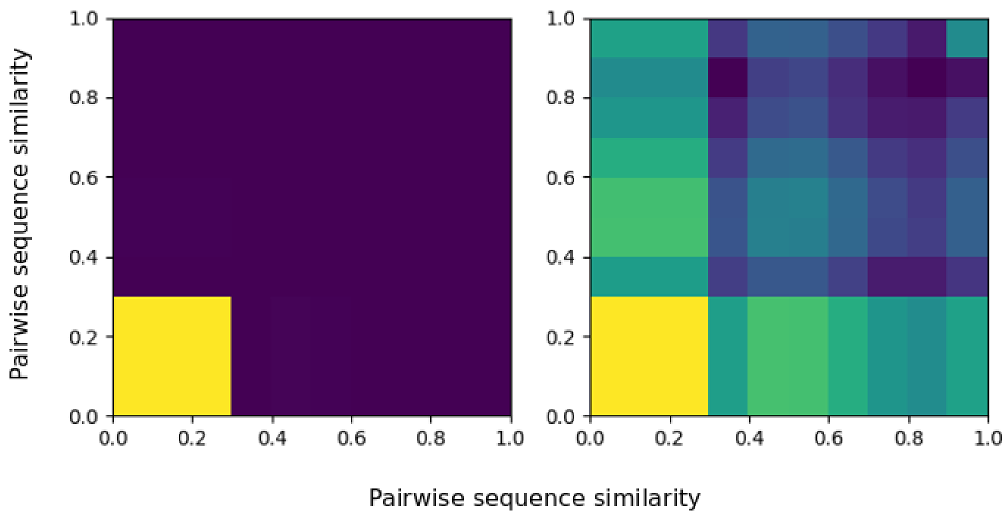

Figure S6: Redundancy analysis between binders in test data. See Table S5 for values.

	0 - 30%	30-40%	40-50%	50-60%	60-70%	70-80%	80-90%	90-100%
0 - 30%	24618884	17412	199895	188349	48543	9988	5104	21649
30-40%	16773	39	172	164	46	7	7	25
40-50%	185493	145	2320	1977	463	73	40	280
50-60%	175907	129	2127	2388	485	84	33	280
60-70%	48105	35	516	594	182	32	18	104
70-80%	10724	9	84	120	21	7	6	35
80-90%	5011	2	41	59	16	4	2	4
90-100%	22613	29	311	307	101	31	6	5071

Table S5: Testing data cross similarities for binders.

Max similarity	Accuracy	AUROC	AUPRC	Sensitivity	Specificity	Precision
0.3	0.8193	0.9063	0.8797	0.6356	0.9433	0.8832
0.4	0.8240	0.9069	0.8811	0.6505	0.9431	0.8870
0.5	0.8391	0.9179	0.9064	0.7539	0.9088	0.8713
0.6	0.8536	0.9278	0.9293	0.8260	0.8806	0.8715

Table S6: Performance of the RF algorithm retrained on datasets filtered for redundancy

Max similarity	Accuracy	AUROC	AUPRC	Sensitivity	Specificity	Precision
0.3	0.8235	0.8798	0.8406	0.7111	0.8903	0.8235
0.4	0.8070	0.8705	0.8287	0.7201	0.8599	0.7580
0.5	0.8179	0.8874	0.8793	0.7107	0.9006	0.8466
0.6	0.7888	0.8593	0.8436	0.7417	0.8251	0.7660

Table S7: Performance of the GNN algorithm retrained on datasets filtered for redundancy

data	binder	threshold	size
test	neg	0.3	4388
test	neg	0.4	4449
test	neg	0.5	4716
test	neg	0.6	4808
test	neg	unfiltered	4817
test	pos	0.3	2961
test	pos	0.4	3053
test	pos	0.5	3861
test	pos	0.6	4713
test	pos	unfiltered	5059
train	neg	0.3	30415
train	neg	0.4	30965
train	neg	0.5	33693
train	neg	0.6	34551
train	neg	unfiltered	34640
train	pos	0.3	17731
train	pos	0.4	18509
train	pos	0.5	25794
train	pos	0.6	33278
train	pos	unfiltered	36423

Table S8: Sizes of the filtered datasets

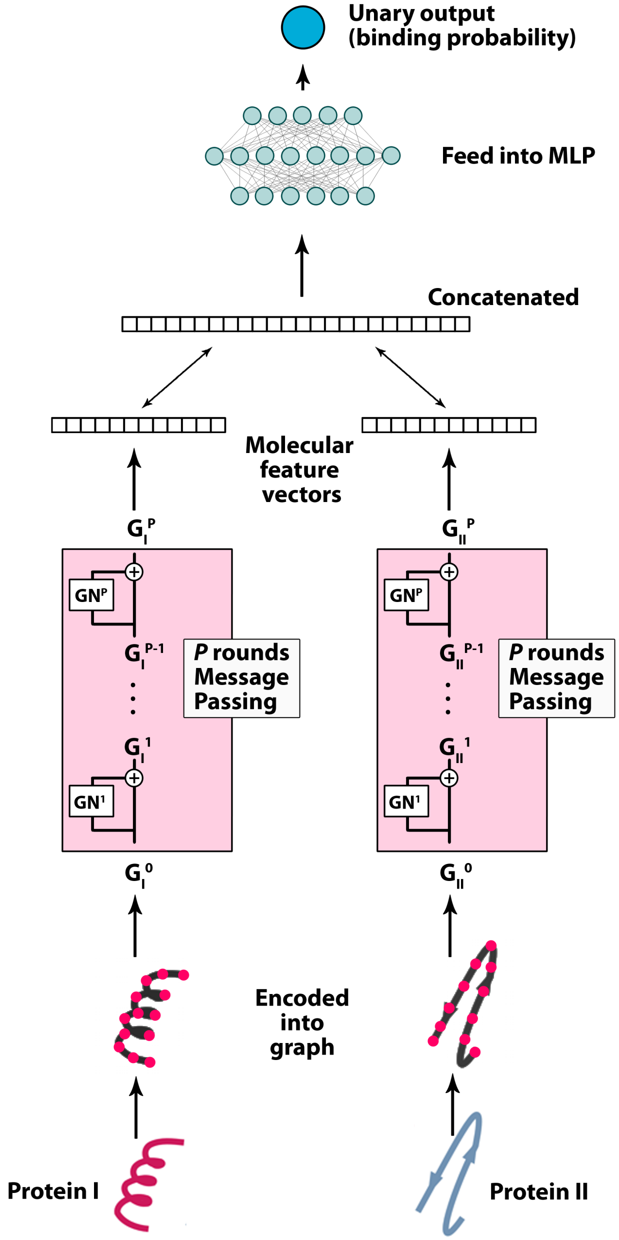

Figure S7: Schematic description of the graph neural network implementation. The proteins are encoded into graphs in which each amino acid is represented by one node and subsequent residues are connected by an edge. During the optimization procedure, the GraphNet (GN) modifies the aggregate and update functions. From each GNN, a feature vector is created. These feature vectors are concatenated and then passed to the final MLP, which predicts the binding. All elements of the entire scheme are optimized commonly in a single back-propagation.

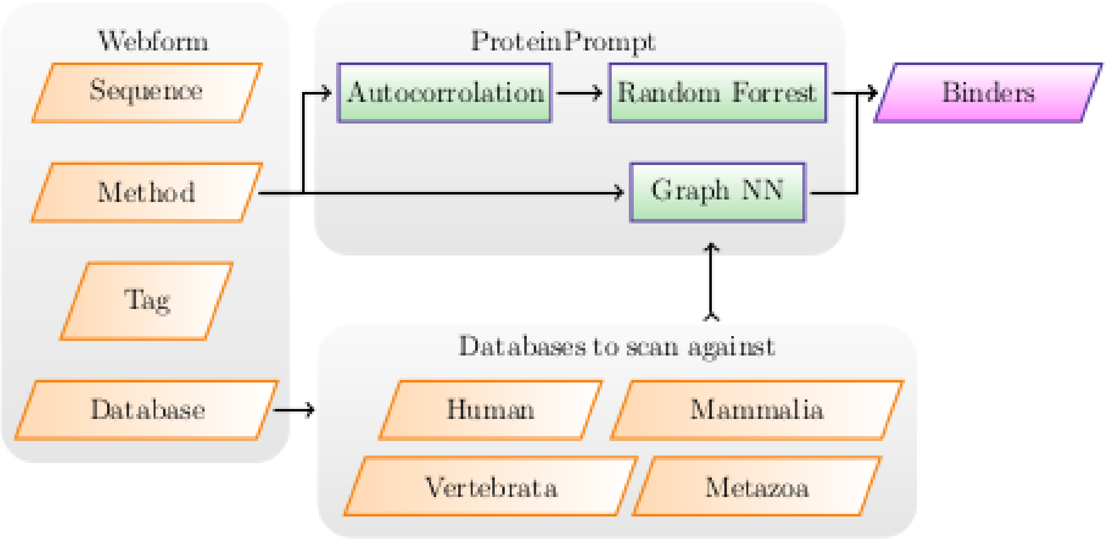

Figure S8: Server workflow. The user pastes the query sequence, provides a unique tag, and selects a method and organism to scan. The server returns a sorted table of potential binders.

3.1.2 Individual test cases

Gene name	AKT1	BRCA1	ERBB2	SRC	STAT1
Protein	RAC-alpha serine/threonine-protein kinase	Breast cancer type 1 susceptibility protein	Receptor tyrosine-protein kinase erbB-2	Proto-oncogene tyrosine-protein kinase Src	Signal transducer and activator of transcription 1-alpha/beta
Uniprot ID	P31749	P38398	P04626	P12931	P42224
Prominent functions	Signal transduction, cell survival	DNA repair, tumor marker	Growth factor receptor, cell proliferation	Proto-oncogene, cell survival	transcription factor, immune response
Cell compartment	cytosol	nucleus	plasma membrane	cytosol	cytosol, nucleus
Number of EV PPIs in STRING.db (high confidence)	22	17	12	9	6
STRING.db score (Mean ± SD)	0.829 ± 0.108	0.822 ± 0.107	0.783 ± 0.041	0.847 ± 0.118	0.772 ± 0.092
Number of EV PPIs found with ProteinPrompt	22	17	12	8	6
ProteinPrompt score (Mean ± SD)	0.903 ± 0.074	0.793 ± 0.138	0.811 ± 0.257	0.905 ± 0.088	0.896 ± 0.129

Table S9: Detailed comparison of the STRING database with ProteinPrompt.

Here we show additional 20 test cases to the 5 presented in 3.1.2 of the main manuscript. The additional proteins were taken from [5]. The procedure was equivalent. Figure S9 is the extended version of Figure 5 in the main manuscript, Table S10 is the extended version of Table S9.

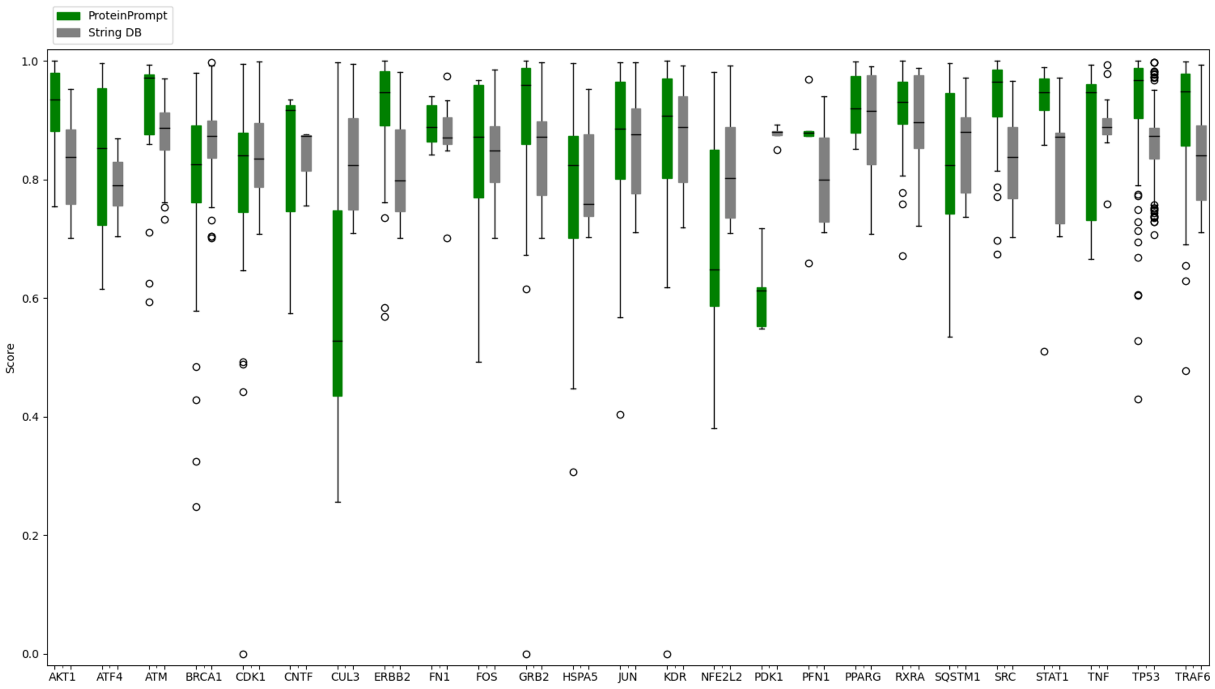

Figure S9: EV PPI scores from ProteinPrompt (green) and from STRING database (grey) for an extended set of 25 different proteins. A few of the EV PPI in the STRING database appear in the training data of ProteinPrompt. Some protein protein pairs that were falsely classified as non binding have been experimentally verified to be binders. This explains the outliers for CDK1, GRB2 and KDR. There are few outliers in the STRING database data due to the cutoff at a confidence score of 0.7.

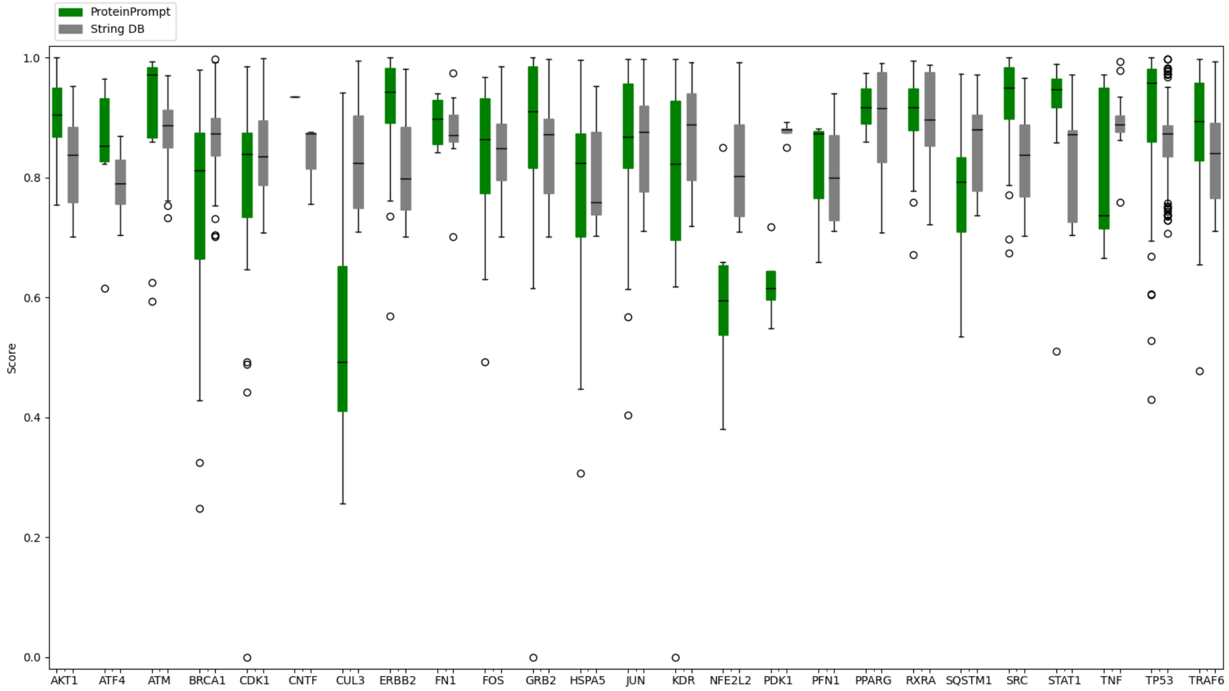

Figure S10: Same as Figure S9 without overlap between STRINGdb and our training data. About 28.5% of the human sequences in STRINGdb were contained in our training data.

Gene Name	AKT1	ATF4	ATM	BRCA1	CDK1	CNTF	CUL3	ERBB2	FN1
Protein	RAC-alpha serine/threonine-protein kinase	Cyclic AMP-dependent transcription factor ATF-4	Serine-protein kinase ATM	Breast cancer type 1 susceptibility protein	Cyclin-dependent kinase 1	Ciliary neurotrophic factor	Cullin-3	Receptor tyrosine-protein kinase erbB-2	Fibronectin
Uniprot ID	P31749	P18848	Q13315	P38398	P06493	P26441	Q13618	P04626	P02751
Function	kinase	transcription regulator	kinase	transcription regulator	kinase	cytokine	enzyme	kinase	enzyme
Cell compartment	cytosol	nucleus	nucleus	nucleus	cytoplasm, mitochondrion, nucleus	cytoplasm	cytoplasm, nucleus	plasma membrane	extracellular matrix
number of experimentally verified PPIs	49	10	18	51	37	3	44	29	7
String DB score (Q1 ... median ... Q3)	0.759 ... 0.838 ... 0.884	0.756 ... 0.79 ... 0.83	0.851 ... 0.888 ... 0.913	0.836 ... 0.873 ... 0.899	0.788 ... 0.835 ... 0.895	0.815 ... 0.874 ... 0.875	0.748 ... 0.824 ... 0.904	0.747 ... 0.798 ... 0.884	0.859 ... 0.871 ... 0.905
number of experimentally verified PPIs found	49	10	18	47	33	3	25	29	7
ProteinPrompt score (Q1 ... median ... Q3)	0.881 ... 0.935 ... 0.98	0.723 ... 0.853 ... 0.954	0.876 ... 0.971 ... 0.977	0.761 ... 0.825 ... 0.891	0.745 ... 0.841 ... 0.879	0.746 ... 0.917 ... 0.926	0.436 ... 0.528 ... 0.748	0.891 ... 0.947 ... 0.983	0.863 ... 0.888 ... 0.926
Gene Name	FOS	GRB2	HSPA5	JUN	KDR	NFE2L2	PDK1	PFN1	PPARG
Protein	Protein c-Fos	Growth factor receptor-bound protein 2	Endoplasmic reticulum chaperone BiP	Transcription factor Jun	Vascular endothelial growth factor receptor 2	Nuclear factor erythroid 2-related factor 2	3-phosphoinositide-dependent protein kinase 1	Profilin-1	Peroxisome proliferator-activated receptor gamma
Uniprot ID	P01100	P62993	P11021	P05412	P35968	Q16236	O15530	P07737	P37231
Function	transcription regulator	kinase	enzyme	transcription regulator	kinase	transcription regulator	kinase	cytoskeleton organization	lig-dependent nuc. receptor
Cell compartment	cytosol, nucleus	cytoplasm, nucleus	cell surface, cytoplasm	nucleus	cell membrane	cytosol, nucleus	cell membrane, cytoplasm, nucleus	cytoskeleton	cytosol, nucleus
number of experimentally verified PPIs	17	74	23	30	21	9	5	5	16
String DB score (Q1 ... median ... Q3)	0.795 ... 0.849 ... 0.89	0.774 ... 0.872 ... 0.898	0.738 ... 0.759 ... 0.876	0.777 ... 0.876 ... 0.92	0.795 ... 0.889 ... 0.941	0.735 ... 0.803 ... 0.889	0.875 ... 0.88 ... 0.882	0.729 ... 0.8 ... 0.871	0.826 ... 0.916 ... 0.976
number of experimentally verified PPIs found	16	73	19	29	20	7	5	5	16
ProteinPrompt score (Q1 ... median ... Q3)	0.769 ... 0.872 ... 0.96	0.86 ... 0.96 ... 0.988	0.702 ... 0.824 ... 0.874	0.801 ... 0.886 ... 0.964	0.803 ... 0.908 ... 0.971	0.587 ... 0.648 ... 0.851	0.552 ... 0.612 ... 0.619	0.873 ... 0.879 ... 0.881	0.879 ... 0.92 ... 0.975
Gene Name	RXRA	SQSTM1	SRC	STAT1	TNF	TP53	TRAF6		
Protein	Retinoic acid receptor RXR-alpha	Sequestosome-1	Proto-oncogene tyrosine-protein kinase Src	Signal transducer and activator of transcription 1-alpha/beta	Tumor necrosis factor	Cellular tumor antigen p53	TNF receptor-associated factor 6		
Uniprot ID	P19793	Q13501	P12931	P42224	P01375	P04637	Q9Y4K3		
Function	lig-dependent nuc. receptor	transcription regulator	kinase	transcription regulator	cytokine	transcription regulator	enzyme		
Cell compartment	cytoplasm, mitochondrion, nucleus	cytosol, nucleus	cytosol	cytosol, nucleus	cell membrane	cytoplasm, mitochondrion matrix, nucleus	cytoplasm, nucleus		
number of experimentally verified PPIs	24	24	46	19	14	118	51		
String DB score (Q1 ... median ... Q3)	0.852 ... 0.896 ... 0.976	0.778 ... 0.881 ... 0.905	0.769 ... 0.838 ... 0.889	0.726 ... 0.872 ... 0.88	0.876 ... 0.888 ... 0.904	0.835 ... 0.873 ... 0.887	0.766 ... 0.841 ... 0.892		
number of experimentally verified PPIs found	24	24	46	19	14	117	50		
ProteinPrompt score (Q1 ... median ... Q3)	0.894 ... 0.931 ... 0.965	0.743 ... 0.825 ... 0.946	0.907 ... 0.965 ... 0.985	0.917 ... 0.947 ... 0.97	0.731 ... 0.947 ... 0.961	0.904 ... 0.967 ... 0.988	0.857 ... 0.949 ... 0.978		

Table S10: Detailed comparison of the STRING database with ProteinPrompt. Only EV PPI from STRING db with a high confidence were used (score above 0.7).

3.1.3 Verification of ProteinPrompt::RF with the datasets of Park and Marcotte

Park & Marcotte [1] performed a detailed analysis of the pairwise input of sequence based protein-protein interaction (PPI) predictions. As a result, the Marcotte lab provides the download of several training and test datasets [3]. In principle, they provide two human and two yeast datasets that differ in their composition: 'human_balanced', 'human_random', 'yeast_balanced', and 'yeast_random'. Each dataset contains 'typical' cross-validation sets (CV) but also sets where the test data was split into distinct classes (C1-C3), depending on the appearance of proteins in the respective training data, see Table S11 for a definition of those test classes. Park & Marcotte also provide the results of 7 different PPI predictions methods, which were trained on that data (M1-M7). Ding *et al* extended this by another 3 methods (MMI, NMBAC, MMI+NMBAC) [2]. The availability of this data has two major benefits: First, when developing a new method for PPI prediction, one can compare directly with the results of these 10 methods without having to go through the often time-consuming process of installing the software and all the necessary dependencies. Second, as in our case, having obtained results with higher accuracy than previous methods it is possible to distinguish whether this increase in prediction quality is due to an improved learning method or whether a more suitable dataset is the cause of the increase.

We performed two tests on each of the four available datasets. First, we ran the normal version of ProteinPrompt, trained on our human training data as described in the main text. The results for the test data in both human subsets 'random' and 'balanced' show a similar level of accuracy than on our own test data. However, it has to be noted that this is not a very robust result, as there may be significant overlap between our training data and the test data provided by Park & Marcotte. Since we have filtered our data very rigorously, as described in the previous section, the verification using our own test data is thus more reliable.

As a second approach, we also trained new random forest models using the training datasets of Park & Marcotte. The models that are summarized as $\text{ProteinPrompt}_{retrain}$ were trained similar to our original random forest model, i.e., we used the given protein pair orientation A-B from the training data file, but also the reversed orientation B-A and two combinations where one of both proteins has the inverted amino acid sequence: A-B_{inv} and B-A_{inv}. This enhancement of the training space has small but consistent positive effects on the models.

Table S12 shows the results for the 'human balanced' dataset, S13 for 'human random', S14 for 'yeast balanced', S15 for 'yeast random'. The first thing to notice is that the accuracy for the human test sets decreases significantly when the random forest model is trained on the Park & Marcotte datasets. However, the achieved AUROC (area under the roc curve) and AUPRC (area under the precision recall curve) values are still highly comparable to the more powerful competitors.

This clearly suggests that our presented combination of autocorrelated biochemical residue scales and a random forest model is as good as other methodological approaches used to predict protein-protein interactions. The true benefit that clearly elevates ProteinPrompt comes with the extensive training and testing datasets that we collected and curated. Here, we could show that collecting as much training data as possible in combination with a rigorous cleaning can boost the prediction accuracy.

The decrease in accuracy for yeast in the normal version of ProteinPrompt, reveals a limitation in generalizability to distant organisms. This is not too surprising since learning methods are generally valid only for the training data domain. We could show that the accuracies increase when models are trained with yeast-specific data.

C1	Both proteins in the test pair are found in the training set
C2	Only one protein in the test pair is found in the training set
C3	Neither protein in the test pair is found in the training set

Table S11: Definitions of C1, C2, C3 from [1]

	AUROC				AUPRC			
Method	CV	C1	C2	C3	CV	C1	C2	C3
ProteinPrompt$_{retrain}$	0.63 ± 0.01	0.64 ± 0.01	0.59 ± 0.01	0.54 ± 0.01	0.67 ± 0.01	0.69 ± 0.01	0.51 ± 0.03	0.54 ± 0.02
ProteinPrompt	0.87 ± 0.01	0.88 ± 0.01	0.77 ± 0.01	0.82 ± 0.01	0.90 ± 0.00	0.90 ± 0.01	0.83 ± 0.01	0.86 ± 0.01
MMI+NMBAC	0.61 ± 0.01	0.62 ± 0.01	0.57 ± 0.02	0.53 ± 0.01	0.64 ± 0.01	0.65 ± 0.01	0.58 ± 0.02	0.53 ± 0.01
MMI	0.61 ± 0.01	0.62 ± 0.01	0.57 ± 0.01	0.53 ± 0.01	0.64 ± 0.01	0.65 ± 0.01	0.58 ± 0.01	0.53 ± 0.01
NMBAC	0.59 ± 0.01	0.60 ± 0.01	0.56 ± 0.01	0.52 ± 0.02	0.62 ± 0.01	0.63 ± 0.01	0.56 ± 0.01	0.52 ± 0.01
M1	0.64 ± 0.01	0.65 ± 0.01	0.61 ± 0.01	0.57 ± 0.02	0.66 ± 0.01	0.67 ± 0.01	0.61 ± 0.02	0.56 ± 0.02
M2	0.59 ± 0.01	0.60 ± 0.01	0.06 ± 0.01	0.57 ± 0.02	0.60 ± 0.01	0.61 ± 0.01	0.60 ± 0.01	0.55 ± 0.01
M3	0.55 ± 0.01	0.56 ± 0.01	0.54 ± 0.01	0.51 ± 0.01	0.60 ± 0.01	0.61 ± 0.01	0.55 ± 0.01	0.51 ± 0.01
M4	0.56 ± 0.01	0.56 ± 0.01	0.54 ± 0.01	0.52 ± 0.01	0.54 ± 0.01	0.54 ± 0.01	0.53 ± 0.01	0.52 ± 0.01
M5	0.59 ± 0.01	0.60 ± 0.01	0.56 ± 0.01	0.53 ± 0.01	0.63 ± 0.01	0.64 ± 0.01	0.57 ± 0.01	0.53 ± 0.01
M7	0.55 ± 0.01	0.55 ± 0.02	0.53 ± 0.01	0.53 ± 0.02	0.55 ± 0.01	0.55 ± 0.01	0.53 ± 0.01	0.54 ± 0.02

Table S12: Comparison of the methods defined in [1, 2] with ProteinPrompt using the test datasets 'human balanced' provided by the Marcotte lab [3].

	AUROC				AUPRC			
Method	CV	C1	C2	C3	CV	C1	C2	C3
ProteinPrompt$_{retrain}$	0.83 ± 0.01	0.83 ± 0.01	0.59 ± 0.01	0.55 ± 0.02	0.84 ± 0.01	0.84 ± 0.01	0.60 ± 0.01	0.55 ± 0.02
ProteinPrompt	0.87 ± 0.01	0.87 ± 0.01	0.77 ± 0.01	0.82 ± 0.01	0.90 ± 0.00	0.90 ± 0.01	0.83 ± 0.01	0.86 ± 0.01
MMI+NMBAC	0.82 ± 0.01	0.82 ± 0.01	0.60 ± 0.01	0.57 ± 0.02	0.83 ± 0.01	0.83 ± 0.01	0.60 ± 0.01	0.56 ± 0.02
MMI	0.81 ± 0.01	0.81 ± 0.01	0.59 ± 0.01	0.56 ± 0.02	0.82 ± 0.01	0.83 ± 0.01	0.59 ± 0.01	0.55 ± 0.01
NMBAC	0.81 ± 0.01	0.82 ± 0.01	0.60 ± 0.01	0.57 ± 0.02	0.83 ± 0.01	0.83 ± 0.01	0.60 ± 0.01	0.56 ± 0.02
M1	0.81 ± 0.01	0.81 ± 0.01	0.61 ± 0.01	0.58 ± 0.03	0.82 ± 0.01	0.82 ± 0.01	0.60 ± 0.01	0.57 ± 0.03
M2	0.85 ± 0.01	0.85 ± 0.01	0.60 ± 0.01	0.58 ± 0.02	0.85 ± 0.01	0.85 ± 0.01	0.60 ± 0.01	0.56 ± 0.02
M3	0.67 ± 0.01	0.68 ± 0.01	0.56 ± 0.01	0.51 ± 0.01	0.68 ± 0.01	0.68 ± 0.01	0.57 ± 0.01	0.51 ± 0.01
M4	0.77 ± 0.01	0.77 ± 0.01	0.57 ± 0.02	0.53 ± 0.02	0.77 ± 0.01	0.77 ± 0.01	0.56 ± 0.01	0.53 ± 0.02
M5	0.81 ± 0.01	0.81 ± 0.01	0.59 ± 0.01	0.54 ± 0.02	0.82 ± 0.01	0.82 ± 0.01	0.59 ± 0.01	0.54 ± 0.02
M6	0.76 ± 0.01	0.77 ± 0.01	0.64 ± 0.01	0.59 ± 0.02	0.79 ± 0.01	0.80 ± 0.01	0.67 ± 0.01	0.60 ± 0.02
M7	0.56 ± 0.01	0.56 ± 0.01	0.53 ± 0.01	0.54 ± 0.03	0.56 ± 0.01	0.56 ± 0.01	0.53 ± 0.01	0.54 ± 0.02

Table S13: Comparison of the methods defined in [1, 2] with ProteinPrompt using the test datasets 'human random' provided by the Marcotte lab [3].

	AUROC				AUPRC			
Method	CV	C1	C2	C3	CV	C1	C2	C3
ProteinPrompt$_{retrain}$	0.67 ± 0.02	0.69 ± 0.01	0.59 ± 0.02	0.53 ± 0.02	0.69 ± 0.02	0.71 ± 0.02	0.59 ± 0.02	0.53 ± 0.02
ProteinPrompt	0.56 ± 0.01	0.56 ± 0.01	0.53 ± 0.02	0.54 ± 0.02	0.55 ± 0.01	0.55 ± 0.01	0.53 ± 0.02	0.54 ± 0.03
MMI+NMBAC	0.65 ± 0.02	0.66 ± 0.02	0.60 ± 0.02	0.55 ± 0.02	0.67 ± 0.02	0.68 ± 0.02	0.60 ± 0.02	0.55 ± 0.02
MMI	0.64 ± 0.02	0.65 ± 0.01	0.60 ± 0.02	0.55 ± 0.02	0.66 ± 0.02	0.68 ± 0.01	0.60 ± 0.02	0.54 ± 0.02
NMBAC	0.63 ± 0.02	0.64 ± 0.02	0.59 ± 0.02	0.54 ± 0.03	0.65 ± 0.02	0.66 ± 0.02	0.59 ± 0.02	0.54 ± 0.02
M1	0.64 ± 0.01	0.64 ± 0.01	0.62 ± 0.02	0.57 ± 0.04	0.65 ± 0.01	0.66 ± 0.01	0.62 ± 0.02	0.57 ± 0.03
M2	0.61 ± 0.01	0.61 ± 0.02	0.62 ± 0.02	0.58 ± 0.03	0.62 ± 0.01	0.61 ± 0.02	0.62 ± 0.02	0.57 ± 0.03
M3	0.55 ± 0.02	0.56 ± 0.02	0.55 ± 0.02	0.51 ± 0.01	0.59 ± 0.01	0.60 ± 0.01	0.56 ± 0.02	0.51 ± 0.02
M4	0.55 ± 0.02	0.55 ± 0.02	0.54 ± 0.02	0.52 ± 0.01	0.53 ± 0.02	0.53 ± 0.01	0.54 ± 0.02	0.52 ± 0.02
M5	0.60 ± 0.02	0.60 ± 0.01	0.55 ± 0.02	0.52 ± 0.02	0.61 ± 0.02	0.62 ± 0.01	0.55 ± 0.02	0.52 ± 0.02
M7	0.55 ± 0.02	0.54 ± 0.01	0.54 ± 0.02	0.53 ± 0.02	0.55 ± 0.02	0.55 ± 0.01	0.54 ± 0.02	0.53 ± 0.02

Table S14: Comparison of the methods defined in [1, 2] with ProteinPrompt using the test datasets 'yeast balanced' provided by the Marcotte lab [3].

	AUROC				AUPRC			
Method	CV	C1	C2	C3	CV	C1	C2	C3
ProteinPrompt$_{retrain}$	0.82 ± 0.01	0.82 ± 0.01	0.58 ± 0.02	0.56 ± 0.03	0.84 ± 0.01	0.84 ± 0.01	0.59 ± 0.02	0.56 ± 0.02
ProteinPrompt	0.56 ± 0.01	0.56 ± 0.01	0.53 ± 0.02	0.54 ± 0.02	0.55 ± 0.01	0.55 ± 0.01	0.53 ± 0.02	0.54 ± 0.03
MMI+NMBAC	0.82 ± 0.02	0.82 ± 0.01	0.62 ± 0.02	0.61 ± 0.02	0.84 ± 0.01	0.84 ± 0.01	0.64 ± 0.02	0.62 ± 0.02
MMI	0.82 ± 0.01	0.82 ± 0.01	0.62 ± 0.02	0.60 ± 0.02	0.84 ± 0.02	0.84 ± 0.01	0.64 ± 0.02	0.61 ± 0.02
NMBAC	0.82 ± 0.01	0.82 ± 0.01	0.61 ± 0.02	0.60 ± 0.03	0.83 ± 0.01	0.83 ± 0.01	0.63 ± 0.03	0.60 ± 0.03
M1	0.82 ± 0.01	0.82 ± 0.01	0.61 ± 0.02	0.58 ± 0.03	0.83 ± 0.02	0.83 ± 0.01	0.62 ± 0.02	0.58 ± 0.03
M2	0.83 ± 0.01	0.84 ± 0.01	0.60 ± 0.02	0.59 ± 0.03	0.84 ± 0.02	0.85 ± 0.01	0.61 ± 0.02	0.58 ± 0.03
M3	0.66 ± 0.02	0.67 ± 0.01	0.56 ± 0.02	0.51 ± 0.01	0.66 ± 0.02	0.66 ± 0.01	0.56 ± 0.02	0.51 ± 0.02
M4	0.76 ± 0.02	0.76 ± 0.02	0.57 ± 0.02	0.54 ± 0.03	0.76 ± 0.02	0.76 ± 0.02	0.58 ± 0.02	0.54 ± 0.03
M5	0.80 ± 0.02	0.80 ± 0.01	0.57 ± 0.01	0.55 ± 0.02	0.81 ± 0.02	0.81 ± 0.02	0.59 ± 0.02	0.55 ± 0.02
M6	0.75 ± 0.02	0.75 ± 0.02	0.59 ± 0.04	0.55 ± 0.03	0.79 ± 0.02	0.79 ± 0.02	0.64 ± 0.03	0.55 ± 0.04
M7	0.58 ± 0.02	0.58 ± 0.01	0.54 ± 0.02	0.53 ± 0.02	0.60 ± 0.02	0.60 ± 0.02	0.55 ± 0.02	0.53 ± 0.02

Table S15: Comparison of the methods defined in [1, 2] with ProteinPrompt using the test datasets 'yeast random' provided by the Marcotte lab [3].

3.4. Feature evaluation

To assess the information content of the feature scales, we calculated the Pearson correlation between all pairs of the scales and attempted to estimate the importance of each scale to the outcome of the models by performing *permutation feature importance*.

3.4.1 Information content of the feature scales

Figure S11 shows the Pearson correlation coefficient between all pairs of scales used to train the RF. Despite some notable correlations, e.g. between hydrophobicity and polarity, hydrophilicity and polarity or surface area and side chain volume, most pairs are not correlated.

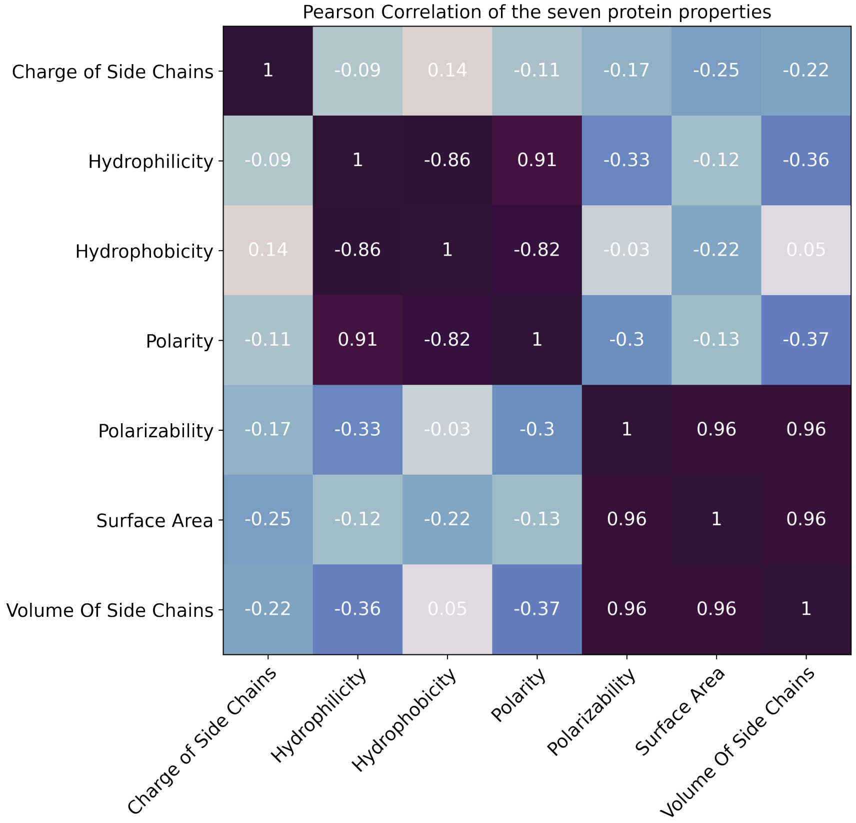

Figure S11: Heatmap of Pearson correlation between different residue scales. Despite some strong correlations between some scales, there is no scale that correlates with all others that could be omitted.

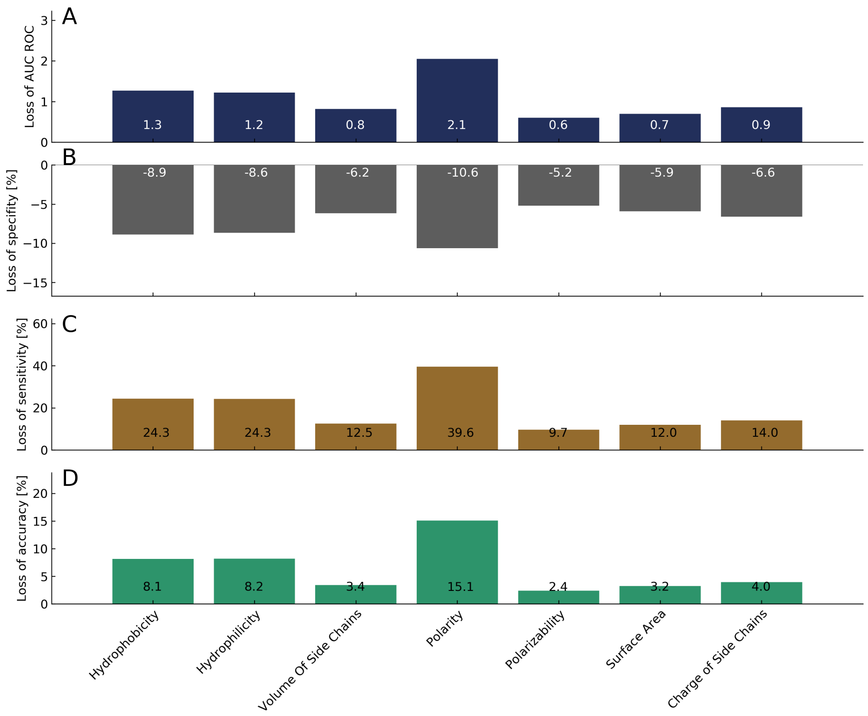

Figure S12: Permutation feature importance analysis for random forest.

3.4.2 Impact of individual scales on RF

To evaluate the importance of each of the seven amino acid scales in our random forest approach, we applied a feature permutation approach as it was illustrated by Breiman [4]. Therein, to introduce noise for a specific feature (amino acid scale) we extracted those 60 corresponding feature vector elements of each protein pair for all pairs in our test set. That means, based on our 420 dimensional feature vector that describes a certain protein pair, we extracted the position 1-30 and 211-240 that represent the 30 autocorrelation values of the hydrophobicity feature for protein one and two. All other features can be selected based on their respective columns in the feature vector. For each of the seven features we therfore extracted a set of 60 autocorrelation values from 9876 protein pairs. We subsequently shuffled those values to guarantee the exact same distribution and re-introduced those randomized feature values in our feature vector.

We ran the model evaluation for each of the seven disturbed feature scales and calculated the difference to our original test data set for the overall accuracy, area under the ROC curve, sensitivity, and specificity. The results can be seen in Figure S12.

In general, we observed a loss of accuracy and AUC for each of the seven features. The sensitivity is also highly decreased for all features while the specificity is alwasy increased. However, the magnitude of gain in specificity is always overturned by the loss in sensitivity, resulting in an overall decrease in accuracy.

The biggest effect could be found for Polarity, which results in a 15% loss of accuracy. Hydrophobicity and Hydrophilicity resulted also in a significant loss in accuracy with slightly over 8%. Minor effects were calculated for the remaining four features with a loss in accuracy between 2.4 and 4.0%.

3.4.3 Impact of the individual scales on GNN

The results of the permutation feature importance are shown in figure S13. Permutation of any scale resulted in large losses of accuracy, sensitivity, specifity or are-under-curve of the receiver operating characteristic.

For this reason, no statement can be made about the significance of the individual scales for the final result. Rather, this is an indicator of the model's lack of robustness to such permutations.

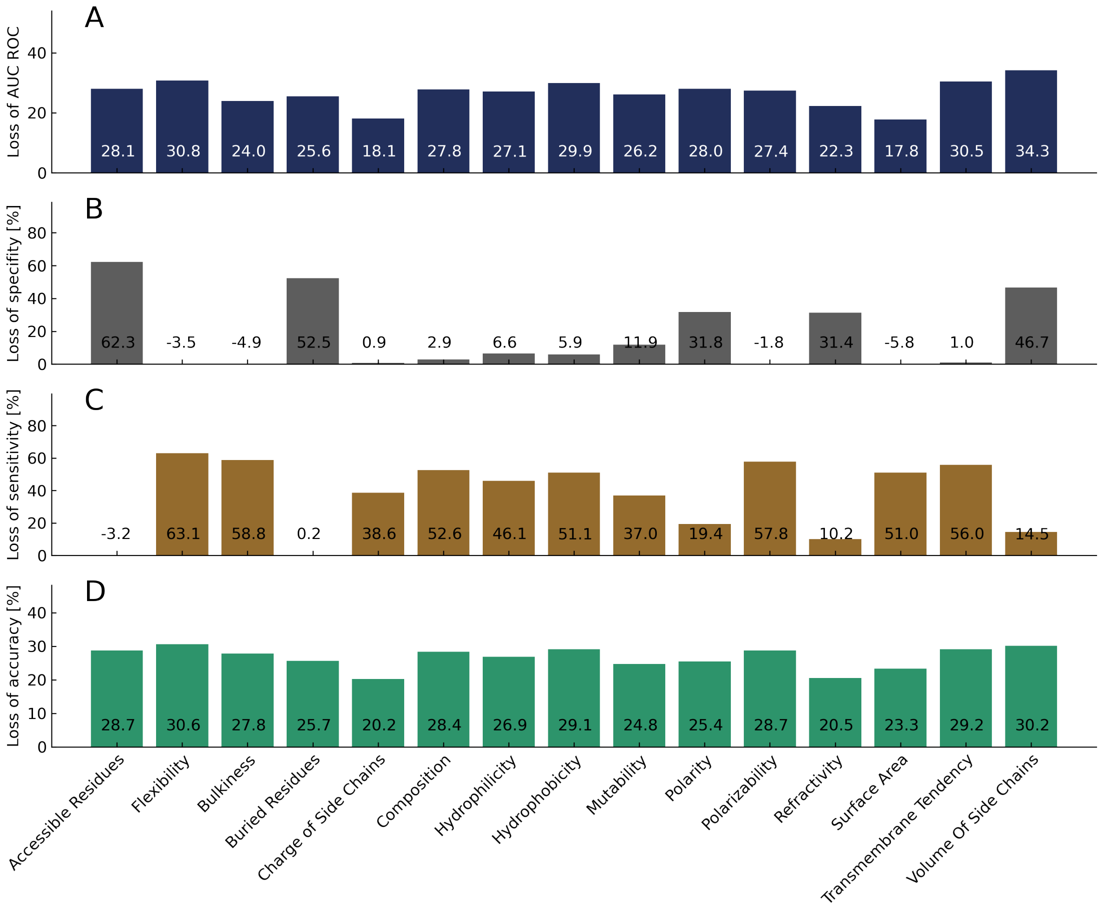

Figure S13: For the GNN model, a permutation analysis of the features was performed to determine the impact of each feature on the model. However, each permutation resulted in an immense loss of accuracy. This is more an indicator that the model lacks robustness to a shift in the input data, and does not allow conclusions about the importance of an individual feature scale.

References

[1] Park, Y. and Marcotte, E. M. (2012). Flaws in evaluation schemes for pair-input computational predictions. *Nat. Methods*, **9**(12), 1134–1136.

[2] Ding, Y., Tang, J., and Guo, F. (2016). Predicting protein-protein interactions via multivariate mutual information of protein sequences. *BMC Bioinformatics*, **17**(1), 398.

[3] Training and test datasets for human and yeast: http://www.marcottelab.org/differentialGeneralization/

[4] Breiman, L. (2001). Random Forests. *Machine Learning*, **45**(1), 5–32 `https://doi.org/10.1023/A:1010933404324`

[5] Dervishi, I., Gozutok, O., Murnan, K., Gautam, M., Heller, D., Bigio, E., and Ozdinler, P. H. (2018). Protein-protein interactions reveal key canonical pathways, upstream regulators, interactome domains, and novel targets in ALS. *Sci Rep*, **8**(1), 14732.
